# The balance of n-6 and n-3 fatty acids in canine, feline, and equine nutrition: exploring sources and the significance of alpha-linolenic acid

**DOI:** 10.1093/jas/skae143

**Published:** 2024-05-22

**Authors:** Scarlett Burron, Taylor Richards, Giovane Krebs, Luciano Trevizan, Alexandra Rankovic, Samantha Hartwig, Wendy Pearson, David W L Ma, Anna K Shoveller

**Affiliations:** Department of Animal Biosciences, University of Guelph, Guelph, ON, CanadaN1G 2W1; Department of Animal Biosciences, University of Guelph, Guelph, ON, CanadaN1G 2W1; Departamento de Zootecnia, Universidade Federal do Rio Grande do Sul, Porto Alegre 91540-000, Rio Grande do Sul, Brazil; Departamento de Zootecnia, Universidade Federal do Rio Grande do Sul, Porto Alegre 91540-000, Rio Grande do Sul, Brazil; Department of Animal Biosciences, University of Guelph, Guelph, ON, CanadaN1G 2W1; Department of Animal Biosciences, University of Guelph, Guelph, ON, CanadaN1G 2W1; Department of Animal Biosciences, University of Guelph, Guelph, ON, CanadaN1G 2W1; Department of Human Health and Nutritional Sciences, University of Guelph, Guelph, ON, CanadaN1G 2W1; Department of Animal Biosciences, University of Guelph, Guelph, ON, CanadaN1G 2W1

**Keywords:** camelina oil, companion animal, flaxseed oil, inflammation, omega-3, omega-6

## Abstract

Both n-6 and n-3 fatty acids (**FA**) have numerous significant physiological roles for mammals. The interplay between these families of FA is of interest in companion animal nutrition due to the influence of the n-6:n-3 FA ratio on the modulation of the inflammatory response in disease management and treatment. As both human and animal diets have shifted to greater consumption of vegetable oils rich in n-6 FA, the supplementation of n-3 FA to canine, feline, and equine diets has been advocated for. Although fish oils are commonly added to supply the long-chain n-3 FA eicosapentaenoic acid (**EPA**), and docosahexaenoic acid (**DHA**), a heavy reliance on this ingredient by the human, pet food, and equine supplement industries is not environmentally sustainable. Instead, sustainable sourcing of plant-based oils rich in n-3 α-linolenic acid (**ALA**), such as flaxseed and camelina oils, emerges as a viable option to support an optimal n-6:n-3 FA ratio. Moreover, ALA may offer health benefits that extend beyond its role as a precursor for endogenous EPA and DHA production. The following review underlines the metabolism and recommendations of n-6 and n-3 FA for dogs, cats, and horses and the ratio between them in promoting optimal health and inflammation management. Additionally, insights into both marine and plant-based n-3 FA sources will be discussed, along with the commercial practicality of using plant oils rich in ALA for the provision of n-3 FA to companion animals.

## Teaser

This review explores the metabolism and recommendations of fatty acids for companion animals, highlighting the significance of n-3 fatty acids and their interplay with n-6 fatty acids for overall animal health. Additionally, sustainable alternatives to conventional fish oil for supplementing n-3 fatty acids are discussed.

## Introduction

It is commonly understood that both n-6 and n-3 fatty acids (**FA**) are important components of mammalian diets that aid in the prevention and management of numerous diseases and are required for normal growth and development (Xenoulis and Steiner, 2010; Bernardi et al., 2012; Sakai et al., 2017; Calder, 2020; DiNicolantonio and O’Keefe, 2020). Research published in the 1950s and 1960s found that substituting saturated fats with vegetable oils with lower levels of saturation (e.g., corn oil, safflower oil, sunflower oil, cottonseed oil) lowered cholesterol in humans (Ahrens Jr et al., 1954; Keys et al., 1957). In addition to the development of technology for large-scale production of vegetable oil at the time, this research encouraged the consumption of greater intakes of unsaturated fats through vegetable oil which are greater in n-6 FA compared to n-3 FA (Simopoulos, 2001). This shifted the dietary intake of n-6 and n-3 FA in humans, and also strongly influenced oil seed production and feed ingredients for animals. The use of n-6 FA-rich oils and grains as feedstuffs for livestock has led to a subsequent increase in the n-6 FA and a decrease in the n-3 FA content in meat and eggs for human consumption, further exacerbating the nutritional imbalances of these FA. Research regarding companion animals emerged in the 1960s and 1970s, with investigations into the safety of feeding common edible oils and leftover cooking oil to dogs (Crampton et al., 1960; Nolen, 1973). Before this, animal scraps and by-products were regarded as the primary fat source for canine diets (Koehn, 1942). Similarly, interest in supplementing oil to equine diets increased in the mid-1970s as a result of the work done by Slade et al. (1975), which suggested that endurance horses fed a diet supplemented with 9% corn oil, a n-6 FA-rich oil, improved athletic performance.

Marine sources are the most rich and efficient vectors of long-chain n-3 FA, eicosapentaenoic acid (**EPA**; 20:5n-3) and docosahexaenoic acid (**DHA**; 22:6n-3), for mammals. Fish oil in particular is the primary commercial source used to supply n-3 FA (Grand View Research, 2020). However, there are concerns that production from global fisheries and aquaculture are not able to adequately sustain the n-3 FA intake and recommendations for intake of the growing human and animal populations. The quantity of aquatic foods, excluding algae, consumed globally has increased more than five times in 60 years (Food and Agricultural Organization [FAO], 2022a). Moreover, since 1961, the global average annual rate of consumption of aquatic foods, excluding algae, has been twice the annual population growth rate (3.0% compared to 1.6%) (FAO, 2022a). The contributions from capture fisheries have remained consistent for the last 30 years, while commercial aquaculture production has allowed for a continuous increase in aquatic food consumption. Additionally, while DHA-rich algal oil is commonly used to fortify commercially available infant formula, as reviewed by Fichtali and Senanayake (2010) and Ochsenreither et al. (2016), algal oil was estimated to make up only 1.8% of the volume of global n-3 FA ingredient market in 2018, according to The Global Organization for EPA and DHA Omega-3s (GOED Omega-3s, 2020). It is important to find alternative sources of n-3 FA that can support the rapidly growing pet food and equine supplement industries, to reduce reliance on fish oil. This review aims to help the pet food industry and those making feeding decisions for companion animals and horses consider the value of including sufficient quantities of n-3 FA. This, in turn, could alleviate some of the discrepancies that may be seen between current “complete and balanced” pet diets, as pertaining to essential or conditionally essential FA. Specifically, focus will be placed on n-6 and n-3 FA recommendations for dogs, cats, and horses, and the practicality of using plant oils rich in α-linolenic acid (**ALA**; 18:3n-3), such as flaxseed, camelina, and canola oil, to increase n-3 FA content of the diet.

## Metabolism and Roles of n-6 and n-3 FA in Companion Animals

Dietary fat is essential in the diets of dogs, cats, and horses as fat provides a concentrated source of caloric energy, enables absorption of fat-soluble vitamins, modulates inflammation and immunity, provides structural integrity of cell membranes, aids in growth and development, and supports skin and coat health (National Research Council, 2006a, 2006b; Xenoulis and Steiner, 2010; Bernardi et al., 2012; Rabionet et al., 2014; Sakai et al., 2017; Calder, 2020; DiNicolantonio and O’Keefe, 2020; Oppedisano et al., 2020).

### Metabolism of n-6 and n-3 FA by companion animals

The polyunsaturated FA linoleic acid (**LA**; 18:2n-6) and ALA cannot be produced endogenously by mammals, and as such, they must be obtained in the diet (Sinclair et al., 2002; Goyens et al., 2006). In the case of dogs and horses, LA can be converted to its long-chain metabolite arachidonic acid (**AA**; 20:4n-6) by elongases and the Δ-5 and Δ-6 desaturase enzymes. In contrast, cats have a limited or null capacity for Δ-6 desaturase activity (Rivers et al., 1975), and thus limited conversion of LA to AA. Although the activity of Δ-5 desaturase was detected, cats maintained on a diet with high concentrations of LA could not maintain AA concentrations in plasma and tissue phospholipids (Trevizan et al., 2012). Although dietary LA may be sufficient to meet the n-6 FA requirements of some cats during adult maintenance (Pawlosky et al., 1994), dietary inclusion of AA is crucial to fulfill the requirements of cats with higher metabolic demands, such as during pregnancy and growth (Pawlosky and Salem, 1996; Pawlosky et al., 1997; Morris, 2004). Similarly, the conversion of ALA to its long-chain metabolite EPA is reliant on the same elongase and desaturase (Δ-5 and Δ-6) enzymes. Subsequently, EPA is converted to the long-chain n-3 FA DHA. While a theoretical argument could be made to suggest that cats may also necessitate pre-formed dietary EPA and DHA at levels comparable to AA due to their limited Δ-6 desaturase activity, no published studies were found to establish a definitive dietary requirement for long-chain n-3 FA for adult cats.

Production of long-chain FA (**LCFA**) in the n-6 and n-3 FA pathways are driven largely by the abundance of their parent FA, LA, and ALA, respectively. Shared use of rate-limiting enzymes in these pathways, ∆-5 and ∆-6 desaturase, creates an interrelated and competitive relationship between these FA families. The most critical enzyme and that responsible for the initial desaturation step from LA and ALA is ∆-6 desaturase, and then subsequent desaturation is mediated by ∆-5 desaturase. A high concentration of n-6 FA competing for elongase and desaturase enzymes allows for more AA produced as opposed to EPA and DHA (Zivkovic et al., 2011). Thus, with the exception of carnivorous mammals such as the cat which have limited ∆ 6 desaturase activity, the relative proportions, in addition to the absolute quantities of both ALA and LA directly determine the net rate of conversion to EPA and DHA, and AA, respectively, as determined by in vitro models (Goyens et al., 2006; Liou et al., 2007). Additionally, Δ-5 and Δ-6 desaturase activity is controlled by dietary intake of n-6 and n-3 FA. Enzymatic activity of hepatic Δ-5 and Δ-6 desaturase in mammals increases during deficiency and is suppressed with increased dietary inclusion of these FA; suggesting that a negative feedback loop is involved in the regulation of these enzymes and the production of AA, EPA, and DHA (Cho et al., 1999a, 1999b).

### The roles of n-6 and n-3 FA in companion animal diets

Both n-6 and n-3 FA are vital for the maintenance of health in companion animals. The prostaglandins and eicosanoids produced in the n-6 and n-3 FA pathways have opposing properties. Eicosanoids produced from AA play a role in platelet activation, immunoactivation, vasoconstriction, and inflammation, whereas EPA and DHA give rise to resolvins, resulting in a pro-resolving, anti-inflammatory effects (Goyens et al., 2006; Liou et al., 2007). Innate inflammation, which is facilitated in part by-products of the n-6 FA series, serves an important role in driving the body’s immune response. However, excessive inflammation contributes to the pathology of many diseases, such as cardiovascular disease, arthritis, asthma, atherosclerosis, cancer, and diabetes. Excessive inflammation can be damaging to tissues, and can often trigger sickness behavior that reduces appetite and growth (Libby et al., 2002; de Visser and Coussens, 2006; Murdoch and Lloyd, 2010; Sorriento and Iaccarino, 2019; Tsalamandris et al., 2019). Beyond its role in supporting inflammation, the n-6 FA are integral for the structure of the epidermis and serve an important role in skin barrier function (Burr and Burr, 1929, 1930; Wertz, 1992).

Although there has been no research demonstrating the definitive need for ALA, EPA, or DHA for maintenance in adult dogs, cats, or horses, the anti-inflammatory effects of n-3 FA have been of interest in helping manage osteoarthritis across all three species (Vandeweerd et al., 2012). A meta-analysis consisting of 72 canine and feline trials investigated the efficacy of several therapeutic diets and nutraceuticals for their effectiveness in the pain management of dogs and cats with osteoarthritis, and presented evidence for clinical analgesic efficacy of dietary n-3 FA (Barbeau-Grégoire et al., 2022). Similarly, the supplementation of n-3 FA to horses has been of particular interest, as joint health and arthritis are two of the most reported reasons for supplement addition or diet modification by horse owners (Hoffman et al., 2009). Indeed, the addition of n-3 FA to the equine diet may improve mobility, reduce markers of inflammation, reduce lameness, and may ultimately improve athletic performance in these animals (Woodward et al., 2007; Manhart et al., 2009; Ross-Jones et al., 2014; Brennan et al., 2017; Caron et al., 2019). Additionally, there is evidence that EPA and DHA fed to adult and senior companion animals, including dogs, cats, and horses, can assist in slowing retinal degeneration, improving cognitive function, regulating the immune system, managing inflammatory airway diseases and improving insulin sensitivity (Lascelles et al., 2010; Bauer, 2011; Hess et al., 2013; Nogradi et al., 2015; Goffin et al., 2017; Pan et al., 2018; Elzinga et al., 2019).

The anti-inflammatory effects of n-3 FA had previously been thought to be only a result of EPA and DHA, but not ALA. Moreover, ALA was only considered important as a precursor to EPA and DHA, or as a source of energy, as reviewed by Stark et al. (2008). However, recent research has shed light on the potential signaling roles of ALA, beyond its function as a precursor to EPA and DHA. Specifically, ALA can directly modulate immune response pathways by activating specific G protein-coupled receptors (**GPCR**s), like G protein-coupled receptor 120 (GPR120), which are involved in regulating inflammatory responses (Hirasawa et al., 2005; Oh et al., 2010; Liu et al., 2022). Activation of these GPCRs by ALA reduces pro-inflammatory cytokine production and attenuates the inflammatory response in various cell types (Wei et al., 2021; Yang et al., 2021).

## Recommendations for n-6 and n-3 FA in Companion Animal Nutrition

When formulating diets, it is important to include an appropriate combination of ingredients to meet the recommended nutrient allowances outlined and to support and maintain the overall health and well-being of companion animals, based on their physiological state. However, there are differing recommendations from regulatory bodies which can lead to confusion as to which specific FA inclusions and ratios should be targeted.

### Scientific and regulatory bodies that provide guidance on companion animal nutrition

The National Research Council (**NRC**) of the USA is a government-funded agency that collects and evaluates research completed by external institutions/industries and uses it to establish minimum nutrient recommendations for various life stages, including growth, reproduction, and adult maintenance (NRC, 2006a). The trade body representing the European pet food industry, the European Pet Food Industry Federation (**FEDIAF**), has produced nutritional guidelines for dogs and cats in the format of a comprehensive review of the NRC data, and other existing science produced, as a practical guide for researchers and manufacturers (FEDIAF, 2021). Similarly, the American Association of Feed Control Officials (**AAFCO**) sets the standards for pet food by creating and revising nutrient profiles based on scientific research but utilizing the recommendations from the NRC as the basis (AAFCO, 2021). In the USA, a pet food can only claim it is “complete and balanced” if it meets or exceeds AAFCO’s minimum recommendations, as outlined in the nutrient profiles. Similarly, pet food products in Europe can only be labeled as “complete and balanced” if they meet or exceed the nutritional guidelines established by FEDIAF, which are based on scientific research and recommendations from the NRC and serve as the European equivalent to AAFCO’s standards in the USA (AAFCO, 2021; FEDIAF, 2021). Although AAFCO and FEDIAF require pet food companies to meet their minimal guidelines for FA provision (Table 1), there is no requirement to list quantities of FA on the labeled nutrient profile of the diet (AAFCO, 2021; FEDIAF, 2021).

**Table 1.** Minimum recommended allowances for ALA, LA, AA, EPA, and DHA defined by the NRC, AAFCO, and FEDIAF

Dog^1^				Cat^1^			Horse
NRC (2006a)	Adult		Growth	Adult		Growth	Adult at maintenance^2^
LA	1.10%		1.30%	0.55%		0.55%	0.50%
AA	—		0.03%	0.006%		0.02%	—
ALA	0.044%		0.08%	—		0.02%	—
EPA + DHA	0.044%		0.05%	0.01%		0.01%	—
AAFCO (2021)	Adult		Growth/reproduction	Adult		Growth/reproduction	—
LA	1.10%		1.30%	0.60%		0.60%	—
AA	—		—	0.02%		0.02%	—
ALA	—		0.08%	—		0.02%	—
EPA + DHA	—		0.05%	—		0.012%	—
n-6:n-3	<30:1		<30:1	—		—	—
FEDIAF (2021) ^*^	Adult		Growth/reproduction	Adult		Growth/reproduction	—
	95 kcal/kg^0.75^	110 kcal/kg^0.75^		75 kcal/kg^0.67^	100 kcal/kg^0.67^		
LA	1.53%	1.32%	1.30%	0.67%	0.50%	0.55%	—
AA	—	—	0.03%	0.008%	0.006%	0.02%	—
ALA	—	—	0.08%	—	—	0.02%	—
EPA + DHA	—	—	0.05%	—	—	0.01%	—

^1^Assumes an energy density of 4,000 kcal ME/kg.

^2^Assuming feed intake of 2% body weight.

^*^kg: kilogram body weight of animal; values are listed as percent of diet on a dry matter basis.

Because the horse’s diet is primarily composed of forage, there are no trade organizations representing or governing the nutritional composition of equine feeds or supplements. Only the NRC has published recommended nutrient intakes for horses (NRC, 2006b).

### Fatty acid recommendations for dogs, cats, and horses

For the n-6 FA, the NRC has published minimum recommendations for LA for both growth and adult life stages in dogs and cats. For AA, recommendations exist for canine and feline growth and feline adult maintenance, but not canine adult maintenance (Table 1). For the n-3 FA, the NRC has set minimum ALA recommendations for growth and adult maintenance in dogs, as well as for feline growth. There are no ALA recommendations for feline adult maintenance. For EPA and DHA, recommendations exist across all lifestages for dogs and cats (NRC, 2006a).

Both FEDIAF and AAFCO have published minimum recommended levels for LA, ALA, EPA, and DHA during growth and reproduction in both dogs and cats. While there are minimum AA recommendations for feline growth and reproduction by FEDIAF and AAFCO, only FEDIAF has a recommended intake for AA for dogs during growth and reproduction. For adult dogs at maintenance, FEDIAF and AAFCO have only set minimum recommendations for LA, and for adult cats at maintenance, only LA and AA (AAFCO, 2021; FEDIAF, 2021). However, a maximum recommended ratio of n-6 FA (LA + AA) to n-3 FA (ALA + EPA + DHA) of 30:1 for canine growth and reproduction and adult life stages has been published by AAFCO (AAFCO, 2021). This ratio informs that the minimum recommended ALA allowance for dogs by AAFCO is the same as that published by the NRC. There is no minimum recommended ratio for dogs and no ratio recommendations at all for cats (Table 1).

While the NRC outlines the same recommended daily intake of LA across all life stages in horses, there are no published recommendations for ALA, AA, EPA, or DHA due to a lack of data (NRC, 2006b). Rather, it is assumed that horses consuming good quality forage in adequate amounts will meet their FA requirements. However, the addition of n-3 FA-rich oils and ingredients, such as flaxseed oil and flaxseed, to equine diets has long been common practice (Warren and Vineyard, 2013). These ingredients have been of interest to horse owners due to their purported effects in producing shiny coats (Goh et al., 2004), and due to the anti-inflammatory effects of n-3 FA, as identified in horses and other mammalian species (Manhart et al., 2009; Caron et al., 2019; Elzinga et al., 2019; Calder, 2020). As such, supplemental n-3 FA and a low dietary n-6:n-3 ratio may be recommended to combat inflammatory states in horses, such as obesity and osteoarthritis.

### Considerations of n-3 FA recommendations

All regulatory councils have outlined similar recommendations for ALA, LA, AA, EPA, and DHA in growing dogs and cats (NRC, 2006a; AAFCO, 2021; FEDIAF, 2021). The need for EPA and DHA in the diet of growing animals for cognitive and visual development, both in utero and after birth has been established for several mammalian species, most notably including humans (Pudelkewicz et al., 1968; Clandinin et al., 1980; Pawlosky et al., 1997; Birch et al., 1998; Delton‐Vandenbroucke et al., 1998). To the authors’ knowledge, there has been no published work specific to DHA intake in growing cats or horses. However, puppies who were fed diets fortified with DHA, or whose dams received supplemental EPA and DHA during pregnancy and lactation had improved vision, memory and learning ability (Heinemann et al., 2005; Bauer et al., 2006; Greco, 2008; Zicker et al., 2012). The current published recommendations for EPA and DHA in the diets of growing puppies and kittens stem from the FA profile of canine and feline milk, respectively, and the current recommendations for human infants (NRC, 2006a).

Currently, recommendations for the inclusion of ALA and pre-formed EPA and DHA, have been made by all three regulatory agencies for growing puppies and kittens, but not for adult maintenance. While both AAFCO and FEDIAF state that there is a growing body of evidence pointing to the potential advantages of n-3 FA, they also state that the available data currently falls short of providing a precise recommendation regarding the appropriate dosage of n-3 FA for adult dogs and cats. Although the NRC does have published guidelines for dietary n-3 FA inclusion for adult dogs and cats, these recommendations are not informed by n-3 FA research done specifically to these species during adulthood. The ALA recommendation for adult dogs at maintenance is informed by the minimum recommendation for LA and the recommendation for ALA during fetal development (NRC, 2006a). The recommendations for EPA and DHA by the NRC for adult dogs are based on data from other species, including humans (NRC, 2006a). For adult cats, the NRC described a lack of data informing n-3 FA requirements and that an intake of 0.01% EPA and DHA for adult cats “appears reasonable”, although it is not explicitly stated why this value was chosen (NRC, 2006a).

Recommendations for n-3 FA inclusion may be necessary for dogs and cats placed on low-fat diets, where very few of these FA may be consumed. Despite evidence supporting the health benefits of n-3 FA inclusion at all life stages, such recommendations have not yet been implemented by AAFCO or FEDIAF. Putting this in the context of pet food production in North America, this leaves the dietary inclusion of n-3 FA for adult diets in the hands of each pet food formulator and likely each independent pet food company.

As there have been no published reports of essential FA (EFA) deficiency in horses, it is assumed that horses consuming good quality forage in adequate amounts will meet their FA requirements. However, if access to forage is limited, or if forage quality is poor, supplementation with ALA and LA should be considered.

## The n-6 to n-3 Ratio

### Current recommendations and research in companion animal nutrition

There is a dearth of data determining the ideal n-6:n-3 FA ratio for companion animals. Existing literature in dogs indicates that a ratio lower than the NRC (<26:1) and AAFCO (<30:1) recommendations should be targeted. In short summation, dietary n-6:n-3 FA ratios of 5.3:1 and 10.4:1 decreased inflammatory markers and increased anti-inflammatory markers in the skin of dogs, as compared to diets with a ratio of 24.1:1 or greater (EPA + DHA contributions to total n-3 FA not listed) (Vaughn et al., 1994). The n-6:n-3 FA ratios of these diets were achieved by varying the quantities of menhaden fish, flax, and safflower oils incorporated into each diet. Kearns et al. (1999) observed that feeding a diet with a lower n-6:n-3 FA ratio of 5:1 compared to a higher ratio of 25:1 (EPA + DHA contributions to total n-3 FA not listed) had a positive effect on the immune status of both young and old dogs, though limited differences in eicosanoid production were observed between dietary groups. These ratios were achieved by increasing the quantities of fish oil and ground flax while reducing chicken fat. However, it is plausible that an n-6:n-3 FA ratio of 5:1 was not low enough to elicit changes in eicosanoid production, as another study found that dogs fed a ratio of 1.4:1 (EPA + DHA: 68.9% total n-3 FA) versus 31:1 (EPA + DHA: <25% total n-3 FA) had a two-fold decrease in inflammatory eicosanoid production in mononuclear cells after stimulation (Wander et al., 1997). Said diets were formulated to be the same, apart from the oil source, which differed between the low (2% fish oil) and high (2% corn oil) n-6:n-3 FA ratio diet. Additionally, a ratio of 1.4:1 was most effective at depressing cell-mediated immunity, though some adverse effects were also observed and will be discussed below. Finally, LeBlanc et al. (2005) saw no adverse effects in 15 healthy hound-cross dogs fed a diet supplemented with menhaden fish oil at a ratio of 3.4:1 (EPA + DHA: 56.1% total n-3 FA) over 12 wk.

Although there is no minimum recommended intake of ALA in adult cats to inform n-6:n-3 FA ratio guidelines, cats exhibited a reduced skin inflammatory response to histamine when supplemented with fish or flaxseed oil on a control diet (formulated with chicken fat), achieving an n-6:n-3 FA ratio of 5:1 (Fish: EPA + DHA: 60.8% total n-3 FA; Flax: EPA + DHA: 5.1% total n-3 FA), compared to those consuming only the control diet at a ratio of 20:1 (EPA + DHA: 10.9% total n-3 FA) (Park et al., 2011). Furthermore, cats consuming a commercial diet supplemented with fish oil (0.7%) and a high AA algae oil (0.2%) at a n-6:n-3 FA ratio of 7.7:1 (EPA + DHA: 61.4% total n-3 FA), as compared to 22.5:1 (EPA + DHA: <15.4% total n-3 FA) had a lower risk of urine stone formation as informed by decreased urine specific gravity, decreased urine calcium concentrations, decreased relative-super-saturation for struvite crystals and greater resistance to oxalate crystal formation (Hall et al., 2017). The physiological impact of the n-6:n-3 FA ratio is likely impacted by a variety of factors, including the age, breed, and workload of said animal (e.g., performance horses), along with the oil source, presence of LCFA, and absolute quantity of oil (Kearns et al., 1999; LeBlanc et al., 2005; Waldron et al., 2012).

Based on the above, there is no clear recommendation that can be made about the ideal n-6:n-3 FA ratio for canine, feline, or equine diets. However, current research supports that at minimum a ratio of below 10:1 should be targeted for dogs and cats. Additionally, the ideal quantities of longer chain (EPA, DHA, and AA) to shorter chain FA (ALA and LA) have not been defined and require consideration, as this ratio may additionally be predictive of the effects that the FA have together.

Due to the increased production and utilization of n-6 FA-rich ingredients, along with AAFCO and NRC having set minimum LA and LA + AA recommendations in canine and feline diets respectively, there is little concern for n-6 FA deficiencies in pet food diets that provide sufficient amounts of crude fat. Similarly, in horses, the consumption of good quality forage in adequate amounts would meet the outlined recommended daily intakes for LA as established by the NRC. However, this rationale could also leave a window for canine, feline, and equine diets that do not provide sufficient n-3 FA, as there are minimal regulatory recommendations for n-3 FA inclusion, nor are n-3 FA-rich oils the most abundantly produced.

There is little research assessing the effects of a low n-6:n-3 FA ratio. However, it could be predicted that based on the LA deficiency symptoms observed in dogs there may also be risks associated with a low n-6:n-3 FA ratio if it were to result in insufficient amounts of LA or AA being utilized by the animal. Possible effects of an excess of n-3 FA in the diet, due to too low of a n-6:n-3 FA ratio, could include an increase in lipid peroxidation, decreased platelet aggregation, or vitamin E deficiency. As mentioned above, and though there were positive impacts of a low n-6:n-3 FA ratio, the dogs fed the 1.4:1 diet also EPA + DHA: 68.9% total n-3 FA) had greater lipid peroxidation (thiobarbituric acid reactive substances) and lower α-tocopherol (vitamin E) concentrations in their plasma, as compared to dogs fed a ratio of 5.4:1 or 31:1 (EPA + DHA: 60.6% and <25% total n-3 FA, respectively) (Wander et al., 1997). A subsequent study also feeding a 1.4:1 diet (EPA + DHA: 69.8% total n-3 FA), but with three concentrations of α-tocopherol supplementation, found that increasing amounts of α-tocopherol supplementation resulted in decreasing amounts of lipid peroxidation (Hall et al., 2002). This indicates that the negative effects of a lower n-6:n-3 FA ratio in terms of lipid peroxidation and vitamin E status can be offset by sufficient α-tocopherol supplementation. A study by LeBlanc et al. (2005) found that supplementation with menhaden fish oil, at a dietary n-6:n-3 FA ratio of 3.4:1 (EPA + DHA: 56.1% total n-3 FA), did not alter platelet aggregation or lipid peroxidation when compared to dogs supplemented with sunflower oil at a dietary ratio of 24:1 (EPA + DHA: <11.98% total n-3 FA), or the same fish oil diet with the addition of vitamin E. In cats, there are conflicting results surrounding the possible effects of a low n-6:n-3 FA ratio on platelet aggregation and function. Adult cats consuming a n-6:n-3 FA dietary ratio of 1.3:1 had altered platelet aggregation and increased bleeding times, as compared to the cats consuming dietary n-6:n-3 FA ratios of 12:1 or 25:1 for 16 wk (EPA + DHA contributions to total n-3 FA not listed) (Saker et al., 1998). The n-6:n-3 FA ratio of the 1.3:1 treatment diet was altered by incorporating greater quantities of menhaden fish oil than corn oil and grease. Large doses of purified EPA (1.69 g) and DHA (0.94 g) fed daily for 4 wk did not inhibit platelet function in healthy adult cats (Bright et al., 1994); however, the FA content of the base diet and the overall dietary n-6:n-3 FA ratio that these cats were receiving were not reported and make it difficult to understand the effects of the whole diet.

## Supplying n-6 and n-3 FA in the Companion Animal Diet

While the practicality of production, cost, and availability of oil ingredients are all factors to note when determining which oil(s) to include in the diets of companion animals, how these ingredients can be used to optimize health and overall well-being must also be considered. As such, understanding how certain ingredients provide benefits (or detriments) at certain inclusions, and in combination with other ingredients, is important to describe and communicate broadly to the pet food and equine feed industries to maximize the health and well-being of dogs, cats, and horses. A single oil cannot be recommended to all companion animals to meet optimal n-6 and n-3 FA requirements for several reasons. Firstly, the optimal requirements of EFA are not well-defined and can vary depending on factors such as the life stage and health conditions of the animal in question. Additionally, the choice of which oil to recommend and the overall n-6:n-3 FA ratio in the diet should be considered in the context of the entire diet formulation.

### Dietary sources of ALA, LA, DHA, EPA, and AA

Dietary oil sources provide different quantities and ratios of n-6 and n-3 FA that are important to consider when formulating canine, feline, and equine diets. More specifically, terrestrial plant-based oils provide rich sources of LA and ALA but do not have the enzymatic capacity to produce FA longer than 18 carbons (Leonard et al., 2004). Plant-based oils rich in ALA include flaxseed, canola, soy, and camelina oil, whereas high concentrations of LA can be found in olive, sunflower, palm, corn, coconut, sesame, safflower, and cottonseed oil (**Table 2**). Although high in ALA, flaxseed, camelina, canola, and soy oil also contain LA, thus have the more balanced n-6:n-3 FA ratio among the different oil varieties. Arachidonic acid, which is only considered essential during growth and reproduction for dogs and essential for cats at all life stages, can be found in animal-derived sources, such as meat, poultry, eggs, and fish (Li et al., 1998). Aquatic sources such as fish (i.e. herring, menhaden, salmon) or fish oils, and algae or algal oils, are all great sources of EPA and DHA. Algae are the foremost source of EPA and DHA in marine ecosystems (Falk-Petersen et al., 1998), and thus provide fish-consuming algae with a direct source of EPA and DHA for our food chain. However, despite their role in mitigating inflammation, EPA and DHA are not considered essential in the diet of adult dogs and cats, due to a lack of studies establishing requirements for these n-3 LCFA.

**Table 2.** Summary of LA, ALA, DHA, and EPA concentrations (% of total FA), LA: ALA ratios, and n-6:n-3 FA ratios in common oil and fat ingredients

	LA	ALA	LA:ALA	AA	DHA	EPA	n-6:n-3^1^
*Vegetable oils* ^2^							
Camelina oil^3^	19.8	35.4	0.56	—	—	—	0.53
Canola oil^3^	18.6	9.14	2.04	—	—	—	1.89
Coconut oil^2^	1.68	0.02	88.4	—	—	—	88.4
Corn oil^2^	53.2	1.16	45.9	—	—	—	45.9
Cottonseed oil^2^	51.4	0.20	257	—	—	—	257
Flaxseed oil, cold pressed^2^	14.3	53.4	0.27	—	—	—	0.27
Olive oil^2^	9.76	0.76	12.8	—	—	—	12.8
Palm kernel oil^2^	1.60	—	>16	—	—	—	>16
Palm oil^2^	9.10	0.20	45.5	—	—	—	45.5
Peanut oil^2^	32.0	—	>320	—	—	—	>320
Safflower oil (LA 70%)^2^	74.1	0.40	185	—	—	—	185
Sesame oil^2^	41.3	0.30	138	—	—	—	138
Soybean oil^2^	50.3	6.54	7.69	—	—	—	7.69
Sunflower oil^2^	65.7	—	>657	—	—	—	>657
Sunflower oil (70% high oleic acid)^2^	3.61	0.19	18.8	—	—	—	18.8
*Animal fats* ^4^							
Beef tallow^2^	3.10	0.60	5.17	—	—	—	5.17
Bacon grease^2^	10.1	1.00	10.2	—	—	—	10.2
Lard^2^	10.2	1.00	10.2	—	—	—	10.2
Poultry fat^4^	19.5	1.00	19.5	—	—	—	19.5
*Marine oils*							
Fish oil herring^5^	1.10	0.80	1.38	0.30	4.90	8.40	0.10
Fish oil menhaden^5^	1.30	0.30	4.33	0.20	9.10	11.0	0.07
Fish oil salmon (sea caught)^5^	1.20	0.60	2.00	0.90	13.8	12.0	0.08
Krill oil^6^	1.67	3.38	0.49	0.20	12.5	19.3	0.05
Algal oil^6^	2.59	0.25	10.4	0.15	51.3	1.03	0.05

^1^n-6:n-3 = (LA + AA):(ALA + EPA + DHA).

^2^Fatty acid profiles sourced from USDA (2023a).

^3^Fatty acid profiles sourced from Burron et al. (2021).

^4^Although neither the USDA or NRC list AA in the nutrient profiles of the animal fats listed in this table, animal fats have been established as a source of AA (Li et al., 1998).

^5^Fatty acid profiles sourced from NRC (2006a).

^6^Fatty acid profiles sourced from Fuller et al. (2020).

^7^Fatty acid profiles sourced from Świątkiewicz et al. (2020).

In addition to oils and fats, other plant and animal-based ingredients, which have not been purified to only oil or fat, will also contribute fat and FA to the diet (Table 3). In canine and feline diets, this includes meat ingredients such as meals and by-products, eggs, as well as grains and pulses to a smaller extent, including corn, oats, lentils, and peas, among many others. In equine diets, the majority of ALA and LA are supplied through forage and supplemental grain concentrates (Table 4). In comparison with grains, most forages contain a very low LA:ALA ratio.

**Table 3.** Summary of LA, ALA, DHA, and EPA concentrations (% of total FA), LA:ALA ratios, and n-6:n-3 FA ratios in commonly used ingredients in commercial canine and feline diets

	LA	ALA	LA:ALA	AA	DHA	EPA	n-6:n-3^1^
*Grains and pulses*							
Barley flour^2^	53.4	5.87	9.10	—	—	—	9.10
Chickpea flour^2^	55.5	2.13	26.1	—	—	—	26.1
Corn gluten^3^	55.5	2.12	26.2	—	—	—	26.2
Corn flour, whole grain, yellow^2^	51.5	1.60	32.2	—	—	—	32.2
Green peas, whole, raw^2^	51.2	12.0	4.27	—	—	—	4.27
Lentils, raw^2^	47.0	12.6	3.73	—	—	—	3.73
Millet flour^2^	62.5	1.08	57.9	—	—	—	57.9
Oat flour^2^	40.7	1.92	21.2	—	—	—	21.2
Rice flour, white^2^	25.7	5.55	4.63	—	—	—	4.63
Rice protein^3^	38.9	1.39	28.0	—	—	—	28.0
Rice, whole^3^	34.6	1.30	26.6	—	—	—	26.6
Sorghum flour^2^	46.7	2.13	21.9	—	—	—	21.9
Wheat flour^3^	58.3	3.41	17.1	—	—	—	17.1
*Meats and animal-based ingredients*							
Beef meal^3^	5.51	0.53	10.4	0.62	0.11	0.02	9.29
Chicken liver, raw^2^	11.4	0.14	81.4	7.79	—	—	137
Chicken meal^3^	14.7	0.60	24.5	1.37	0.11	0.02	22.0
Egg, whole, dried^2^	17.3	0.42	41.2	1.76	0.49	—	21.0
Lamb meal^3^	2.01	1.05	1.91	0.62	0.23	0.21	1.77
Turkey, ground, raw^2^	27.1	1.48	18.3	1.41	0.12	0.09	16.9
Turkey liver, raw^2^	26.7	0.91	29.3	9.00	1.07	0.21	16.3
*Fish*							
Atlantic salmon, farmed, raw^2^	8.40	1.40	6.00	0.86	10.3	8.03	0.47
Herring meal^3^	4.48	0.94	4.77	0.67	11.3	5.49	0.29
Whitefish, mixed species, raw^2^	5.34	3.56	1.5	4.41	18.6	6.33	0.34

^1^n-6:n-3 = (LA + AA):(ALA + EPA + DHA).

^2^Fatty acid profiles sourced from U.S. Department of Agriculture (2023a).

^3^Fatty acid profiles sourced from NRC (2006a).

**Table 4.** Summary of LA and ALA composition (% of total FA) and n-6:n-3 FA ratios in forages and grain concentrates commonly fed to horses in North America

Feedstuff	LA	ALA	n-6:n-3^1^
*Forages*			
Fresh Orchardgrass^2^	15.7	51.8	0.30
Bermuda Hay^3^	18.8	20.7	0.91
Timothy Hay^3^	18.8	50.6	0.37
Fresh Alfalfa^1^	19.9	41.7	0.48
Alfalfa Hay^3^	18.5	36.8	0.50
Pasture (Mixed)^3^	18.2	53.8	0.34
Alfalfa Silage^2^	18.2	32.2	0.57
*Concentrates* ^3^			
Oats	42.0	1.80	23.3
Beet pulp	49.8	6.30	7.90
Canola meal	31.1	7.60	4.09
Soybean meal	54.2	8.40	6.45
Rice bran	38.1	1.50	25.4
Barley grain	55.9	4.30	13.0
Cracked corn	55.7	1.60	34.8
Linseed meal	14.2	56.0	0.25

^1^n-6:n-3 = LA:ALA.

^2^Fatty acid profiles sourced from Glasser et al. (2013).

^3^Fatty acid profiles sourced from Mad Barn (2023).

The effects of oxidation on the FA profile of an ingredient must also be considered as the FA profile can change with shelf life, depending on how the ingredient has been stored (Crapiste et al., 1999; Zhang et al., 2013). Research by Zhang et al. (2013) reported that the relative concentration of ALA in flaxseed oil decreased while LA increased with oxidation. The change in ALA was more significant than that of LA because ALA is more susceptible to oxidation. Although the authors published the regression equations between the FA and heating time, the specific numerical quantities nor the ratios between these FA were reported.

### Supplying ALA in the diet

When considering the n-6:n-3 FA or the LA:ALA ratio of companion animal diets it is important to find additional sources to increase the inclusion of n-3 FA to subsequently lower the n-6:n-3 FA ratio. These additional n-3 FA can be provided in the form of an ALA-rich plant-based oil (e.g., flaxseed or camelina oil) assuming the DHA, and to a lesser extent, the EPA requirements for the animal are already being met. Although ALA is not capable of maintaining EPA and DHA concentrations in the tissues at equivalent concentrations as marine sources (Dominguez et al., 2021), there is a lack of research looking at the use of marine-based sources in conjunction with other plant-based oil sources in companion animals. A review of the individual effects of ALA, EPA, and DHA suggested that the beneficial effects of ALA on inflammation may be independent of its conversion to EPA and DHA (Anderson and Ma, 2009). Hence, combining marine-based and plant-based oil sources has the potential to create additive beneficial effects; however, further research is needed to fully explore the separate role of ALA vs. ALA + DHA + EPA on the physiological response in animals in different physiological states.

Research on individual ingredients provides significant contributions to the body of knowledge on nutrition and metabolism; however, it often does not consider the practical and economic factors that must be examined when formulating and producing a commercial diet or supplement. When formulating diets and oil supplements, formulators can use a variety of oil or fat sources to ensure that both the requirements and recommendations for FA of the animal are being met, while still considering the practicality and economic factors associated with each ingredient.

## Practical Considerations of Different n-3 FA Oil Sources

### Vegetable oils

#### Changes in vegetable oil production over time

**Figure 1.**
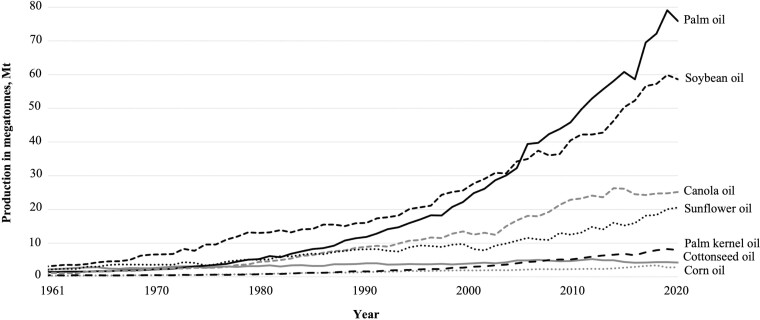
Global production of major vegetable oil types from 1961 to 2020 based on data published by the FAO (2022b).

**Figure 2.**
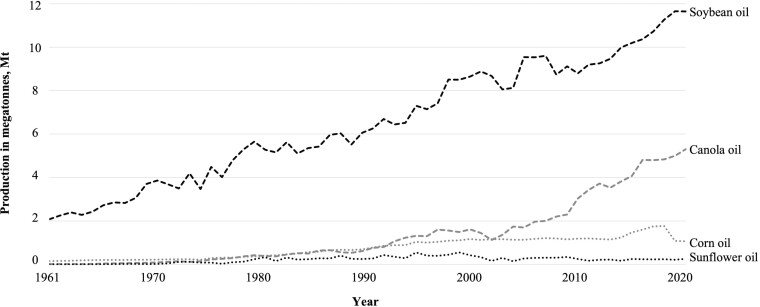
North American production from major vegetable oil types from 1961 to 2020 based on data published by the FAO (2022b).

Global oil production for use in animal and human diets has increased significantly over the last century (Figure 1). Specifically, soybean oil production has grown dramatically since its introduction into North America in the 1970s due to its flexibility in growing conditions, high yield, and low production costs (Figure 2; US Department of Agriculture [USDA], 2023b). Soon after its introduction, soybean production began dominating the oil crop sector due to an increased demand for soybean meal inclusion in animal feeds, biofuels, and vegetable oils, creating a subsequent decrease in the proportional diversity of oilseed crops produced. Oil production has followed an upward trend over the past decade and continues to be impacted by global demand and national policies regarding product labeling and genetically modified organisms (**GMOs**), causing minor changes to the proportional diversity of oilseed crops grown and supplied to humans and animal nutrition sectors.

#### Vegetable oil production and availability today

The demand for oil ingredients represented by the pet food, equine feed, and supplement industries alone does not represent a significant enough proportion of overall demand to influence the cost of oil. However, demand from other industries such as human food, livestock feed, and biofuels significantly drives the price of oil. As such, the oil sources used for pet and equine food formulations are impacted by the demand in these other markets, making the analysis of these industry trends relevant to the current discussion. Additionally, military conflicts and wars can have a significant impact on global trade and the global economy.

Palm oil is the most widely traded vegetable oil globally, with demand projected to increase substantially in the future (Vijay et al., 2016). As palm requires a warm and humid environment to grow (Oettli et al., 2018), Indonesia and Malaysia are the two largest producers of palm oil globally; together contributing approximately 85% of the world’s global palm oil production (USDA, 2023c). However, the North American palm oil market is estimated to grow at a compounded annual growth rate of 7.5%, reaching USD 32.7 billion by 2027 from 22.5 billion in 2021 (Market Data Forecast, 2023). Soybean oil dominates the oilseed sector in North America and is the second most produced oil type globally due to its use in the food processing industry (FAO, 2022b). The production of soybean oil accounts for approximately 90% of US oilseed production and is expected to be increasingly used for biofuel production in the coming years (USDA, 2023b). The global production of canola species (*Brassica napus, Brassic rapa,* and *Brassica juncea* of canola quality) contributes to roughly 15% of total vegetable oil production, making it the third most important crop globally for oil production after soybean oil and palm oil, respectively (Mcvetty et al., 2016; FAO, 2022b). Canada remains the largest exporter of canola products globally, contributing approximately 18% of global canola oil production (FAO, 2022b). In 2022, Canada exported a total of 2.6 million metric tonnes of canola oil, valued at CAD 6.2 billion (Vijay et al., 2016). Global sunflower oil production reached 19.1 million metric tons in the 2020/21 marketing year, accounting for 9% of global vegetable oil production (206.5 million tons) (USDA, 2023b). Sunflower oil is the fourth most produced oil globally, with Ukraine and Russia historically being the first and second largest producers, respectively. In 2019 and 2020, these two countries together accounted for 56% and 58% of international sunflower oil production, respectively (FAO, 2022b). The Russia-Ukraine conflict resulted in sunflower oil prices increasing by 36% between February 2022 to May 2022 (International Monetary Fund, 2023), as supply chains and exports were immediately disturbed and panic buying ensued (Berkhout et al., 2022). Although prices have since decreased (International Monetary Fund, 2023), there is concern that future supplies will be affected due to reduced seeding and harvest, loss of land, and displaced farmers during the conflict (Hellvig and Blanaru, 2023; Sobczak-Malitka and Sobczak, 2023).

In addition to these major and largely produced oil crops, North America is a large trade representative of minor oil crops rich in n-3 FA that have become popular for use in companion animal diets, such as flaxseed and camelina. Specifically, Canada is the largest trade representative of flaxseed grains and fibers (FAO, 2022b). However, flaxseed is still considered a minor crop in Canada and does not compare to the production of canola or soybeans (Agriculture and Agri-Food Canada, 2022; FAO, 2022b). Camelina oil is a niche oilseed crop that is primarily grown in the Canadian prairie provinces, with approximately 10,000 acres seeded in 2020 (Eynck et al., 2021).

#### Genetically modified organisms

Consumers have negative associations with GMOs versus non-GMOs, and worldwide, consumers display limited understanding and misconceptions concerning genetic modification (Lefebvre et al., 2019). In a survey done by Schleicher et al. (2019), respondents ranked “Organic” and “GMO-Free” as being more important characteristics of pet foods than appearance, packaging, or color. Additionally, consumers preferred dog food with increasing amounts of organic ingredients, which cannot be GMO (Simonsen et al., 2014). Consumer perceptions are worth considering when deciding on an oil for a feed or supplement formulation as many of the highest-producing, and most utilized, oilseed crops are GMOs. For example, 81% of all soybeans planted and 88% of corn planted in Canada in 2023 were GMOs. Additionally, GMO canola made up 95% of canola planted in Canada  (USDA, 2023). Some non-GMO plant-based oils include sunflower, safflower, olive, flaxseed, camelina, and coconut oils which all have distinct benefits for canine diets; however, flaxseed and camelina oil are the most abundant sources of ALA.

### Marine sources and considerations for environmental sustainability

Fish oil is the most commonly used ingredient to supply EPA and DHA in a diet. The concentrations of EPA and DHA in fish oils can vary widely depending on many factors including the species, diet and age of the fish, the geographical location, season, and the processing methods used (Kirsch et al., 1998; Cordier et al., 2002; Luzia et al., 2003; Ibarz et al., 2005; Guler et al., 2007; Gladyshev et al., 2018). Typical EPA and DHA concentrations in fish oils range from 5% to 18% and 6% to 25%, respectively, as reviewed by Racine and Deckelbaum (2007). However, the annual global supply of fish from current marine fish stocks is, unfortunately, unable to meet the required dietary EPA and DHA intake that the World Health Organization has set for humans, let alone livestock and companion animals (Tocher, 2015; Betancor et al., 2017; West et al., 2019). Additionally, projections of current fish stocks estimate the collapse of nearly all commercially exploited fish stocks by mid-century (Pauly et al., 2003; Worm et al., 2006).

Commercial fish practices also raise ethical concerns related to worker conditions, such as forced labor, low wages, and hazardous working conditions (Jin et al., 2001; Mileski et al., 2020). These issues are often more prevalent in low-income countries with less enforcement of these regulations (Vandergeest, 2018; International Labour Organization, 2023). Environmental concerns associated with commercial fishing practices include overfishing, harm to marine life, bycatch, habitat destruction, and pollution in aquatic environments (Henwood and Stuntz, 1987; Chan et al., 1988; Auster et al., 1996; Harrington et al., 2005; Farmaki et al., 2014; Wang et al., 2020).

Aquatic foods are one of the most globally traded commodities (FAO, 2022a), and processing fish for their oil generally requires more energy and has a greater environmental footprint compared to vegetable oil production. While vegetable oil production can have varying environmental impacts depending on factors such as growing location and crop management (Beyer and Rademacher, 2021) , fish oil production tends to have a higher environmental impact for several reasons. This is due to the energy-intensive nature of harvesting and transporting the fish, oil extraction, and refrigeration (Mitchell and Cleveland, 1993). While the total global emissions generated by aquaculture are on average greater than that of oil crops, the emissions are still less than those produced by livestock production (i.e. dairy and beef cattle, swine, chickens, etc.) (MacLeod et al., 2019). However, aquaculture tends to require greater energy inputs than livestock production (Hilborn et al., 2018).

As such, to achieve environmental sustainability within the pet food industry, there needs to be a shift away from heavy reliance on fish oils to supply n-3 FA to dogs and cats. In fact, the current recommendations for EPA and DHA are quite low in dogs and cats, so meeting these recommendations with a lower inclusion of fish oil, or more ideally the inclusion of algal oil, would help to support the environmental sustainability of dog and cat food manufacturing. Algae serve as the primary sources of EPA and DHA in the marine ecosystem, and fish obtain EPA and DHA by consuming algae directly or by consuming other fish that have fed on algae (Ackman et al., 1964). The algae processed for algal oil is grown commercially in controlled environments and thus is considered more sustainable than traditional fish oil as it does not deplete fish populations or cause harm to marine ecosystems or other marine species (Beal et al., 2018). Algae also has a high oil content. One hectare of algae can produce 90,000 L of oil, compared to canola and soybean at 1,200 L and 450 L, respectively (Christi, 2007). The concentrations of EPA and DHA in commercial algal oil range from 0.77% to 1.51% and 23.8% to 42.4%, respectively (Kleiner et al., 2015). However, the concentrations of EPA and DHA in algae vary depending on the strain of algae and the cultivation conditions, as reviewed by Sijtsma and de Swaaf (2004) and Gladyshev et al. (2013).

Canine, feline, and equine diets and supplements need to contain sufficient n-3 FA-rich oil sources that are environmentally sustainable to support an appropriate endogenous n-6:n-3 FA ratio. For dogs and horses in particular, this can be done by providing plant-based oil sources rich in ALA, as this will not only provide ALA as an essential nutrient, but there will also be a certain amount of conversion of ALA to EPA, and to a limited extent, DHA (Bauer, 2011). Additionally, as horses are herbivores, supplementing fish or algal oils as sources of n-3 FA may result in food refusal and problems with palatability (Hess et al., 2012), unless these oils are deodorized to reduce the olfactory component of fish and algal oils.

## Conclusion

Increased demand and consumption of processed foods, along with globalization, industrialization, and population growth, have all been contributing factors to the increased production of n-6, rather than n-3, FA-rich oil products in North America. The pet food industry’s dependence on the market driving prices and product availability has thus resulted in increased incorporation of n-6 FA-rich ingredients in canine and feline diets. While n-6 FA are essential for normal physiological function in dogs and cats, a high n-6:n-3 FA ratio will foster a pro-inflammatory state in the body that supersedes natural, innate inflammation. Therefore, including ingredients rich in n-3 FA is necessary to allow for an endogenous balance of these pro- and anti-inflammatory promoting FA pathways. Factors employed by the pet food and equine supplement industry in determining which n-6 or n-3 FA-rich ingredients to use should include an assessment of the FA profile of each ingredient, with heavy consideration of both the absolute quantity of each FA in the diet and/or supplement as well as the final n-6:n-3 FA ratio of the diet and/or supplement being formulated. Additional consideration for consumer preferences and economic, environmental, and social sustainability must be taken into account for ingredient selection. In total, a reliance on a single lipid source will not be sufficient in meeting the essential and conditionally essential FA recommendations of a dog or cat, but rather oil(s) rich in both LA and ALA should be provided. Provided in sufficient quantity, LA and ALA will support the endogenous production of AA and EPA in dogs and horses, so that additional supplementation is only needed during certain life stages, such as growth and reproduction or in certain disease states that may require EPA and DHA to reduce inflammation or support development. However, in comparison, AA is considered essential for cats as a result of low Δ-6 desaturase activity. Regardless, an ingredient rich in EPA and DHA, such as fish or algal oil, should be included in a quantity sufficient to meet the DHA, and to a lesser extent EPA, recommendations. While in general, more data is needed to optimize the diets of dogs, cats, and horses to meet the needs for growth, maintenance, reproduction, and prevention of chronic diseases, the inclusion of EPA or DHA-rich ingredients in excess is not warranted given the practical limitations at this time in terms of sustainability.

## Abbreviations

AA: arachidonic acid
AAFCO: Association of American Feed Control Officials
ALA: α-linolenic acid
DHA: docosahexaenoic acid
EFA: essential fatty acid
EPA: eicosapentaenoic acid
FA: fatty acids
FAO: Food and Agricultural Organization
FEDIAF: European Pet Food Industry Federation
LA: linoleic acid
LCFA: long-chain fatty acid
NRC: National Research Council
